# Changes in Antioxidative, Oxidoreductive and Detoxification Enzymes during Development of Aphids and Temperature Increase

**DOI:** 10.3390/antiox10081181

**Published:** 2021-07-25

**Authors:** Roma Durak, Jan Dampc, Monika Kula-Maximenko, Mateusz Mołoń, Tomasz Durak

**Affiliations:** 1 Institute of Biology and Biotechnology, University of Rzeszów, Pigonia 1, 35-310 Rzeszów, Poland; jdampc@ur.edu.pl (J.D.); mateuszmolon@univ.rzeszow.pl (M.M.); tdurak@univ.rzeszow.pl (T.D.); 2 The Franciszek Górski Institute of Plant Physiology, Polish Academy of Sciences, Niezapominajek 21, 30-239 Kraków, Poland; m.kula@ifr-pan.edu.pl

**Keywords:** aphids, enzymatic markers, oxidative stress, developmental stages

## Abstract

Temperature, being the main factor that has an influence on insects, causes changes in their development, reproduction, winter survival, life cycles, migration timing, and population dynamics. The effects of stress caused by a temperature increase on insects may depend on many factors, such as the frequency, amplitude, duration of the stress, sex, or the developmental stage of the insect. The aim of the study was to determine the differences in the enzymatic activity of nymphs and adult aphids *Aphis pomi*, *Macrosiphum rosae* and *Cinara cupressi*, and changes in their response to a temperature increase from 20 to 28 °C. The activity of enzymatic markers (superoxide dismutase (SOD), catalase (CAT), glutathione S-transferase (GST), β-glucosidase, polyphenol oxidase (PPO) and peroxidase (POD)) in aphid tissues was analysed for three constant temperatures. The results of our research showed that the enzymatic activity of aphids (measured as the activity of antioxidant, detoxifying and oxidoreductive enzymes) was mainly determined by the type of morph. We observed a strong positive correlation between the activity of the detoxifying and oxidoreductive enzymes and aphids’ development, and a negative correlation between the activity of the antioxidant enzymes and aphids’ development. Moreover, the study showed that an increase in temperature caused changes in enzyme activity (especially SOD, CAT and β-glucosidase), which was highest at 28 °C, in both nymphs and adults. Additionally, a strong positive correlation between metabolic activity (heat flow measured by microcalorimeter) and longevity was observed, which confirmed the relationship between these characteristics of aphids. The antioxidant enzyme system is more efficient in aphid nymphs, and during aphid development the activity of antioxidant enzymes decreases. The antioxidant enzyme system in aphids appears to deliver effective protection for nymphs and adults under stressful conditions, such as high temperatures.

## 1. Introduction

Increasing ambient temperature can cause changes in insect biology, behaviour and development, and also changes at cellular and metabolic levels, disrupting the natural balance between ROS production and reduction [1,2,3,4,5]. Oxidative stress (OS) is caused by an excessive amount of ROS in an insect’s body, the generation of which is increased due to stresses such as thermal stress. Temperature affects the intensity of respiration and the rate of ROS formation, the amount of which increases with the consumption of oxygen by organisms [6,7,8,9,10,11]. In order to defend against the effects of ROS, organisms have a number of defense mechanisms [12]. Herbivores, such as chewing insects [6,13,14,15] and sucking-piercing insects, which include aphids [16,17,18,19,20], possess a system of enzymes, the role of which is to destroy toxic ROS.

Aphids develop physiological strategies to adapt to stress factors and one of the most rapid responses to abiotic stress is their enzymatic system—a rapid cascade of interacting antioxidative, oxidoreductive and detoxification enzymes [6,19,21,22,23]. The main antioxidant enzymes, which appear in all aerobic organisms and are responsible for the neutralisation of ROS, are superoxide dismutase (SOD) and catalase (CAT) [20,24]. SOD catalyses the decomposition of the superoxide radical into oxygen and hydrogen peroxide, thus preventing the formation of toxic hydroxyl radicals [7]. CAT catalyses the reactions of the decomposition of H_2_O_2_ into molecular oxygen and water. In aphids, this occurs mainly in the midgut, the middle part of the digestive system, which is the “centre” of H_2_O_2_ production [18,25]. Detoxification enzymes, β-glucosidase and glutathione S-transferase (GST) play an important role in the insect’s resistance to secondary metabolites of the host plants and other xenobiotics [26]. GST are a group of multifunctional enzymes that catalyse the conjugation of glutathione with a wide range of endogenous compounds and xenobiotics for detoxification, protection against oxidative damage, isomerisation, and intracellular transport [27]. β-glucosidase hydrolyses the breakdown of o-glycosyl bonds; activity of this enzyme is strongly related to the chemical composition of the host plant [28]. Environmental stresses can lead to a chemical modification of the phloem sap, which leads to a drop in glucose levels, resulting in the displacement and initiation of feeding by the aphids in the mesophyll cells. The aphids begin to take up other sugars, such as sucrose, which are dehydrolysed by β-glucosidase to glucose and fructose. Under stress, the phloem probing phases are disturbed, which leads to the take up of more sucrose by the aphids [29]. The main task of oxidoreductive enzymes (which include, for example, polyphenol oxidase (PPO) and peroxidase (POD)) is to minimise plant defense mechanisms targeting herbivorous insects, by reducing plant phenolic compounds and their derivatives taken with plant nourishment [30]. The mechanism of the enzymatic defense system allows insects to neutralise the effects of OS in tissues [6]. Temperature may also affect the lifespan of insects by changing their energy status and the generation of molecular damage [31,32]. Due to the coordinated interaction of antioxidative, oxidoreductive and detoxification enzymes, and an increase in metabolic rate, the insects can protect themselves against excessive induction of ROS during high temperatures [33] The consequence of this may be a shortening of the insect’s lifespan [32].

Aphids (Hemiptera, Aphidoidea) are herbivores with the status of major pests for both crops and ornamental plants. *Aphis pomi* (De Geer, 1773) is an oligophagous aphid that feeds on trees and shrubs of the Rosaceae family such as apple, pear and quince. The species often forms large, strong honey-dewing colonies, which causes leaves to curl. *Macrosiphum rosae,* the rose aphid, (Linnaeus, 1758) is also an oligophagous that feeds on plants of the Rosaceae family, but particularly on decorative plants. *Cinara* (*Cupressobium*) *cupressi* (Buckton, 1881), the cypress aphid, is a voracious pest of *Cupressus* spp. and other Cupressaceae, for example *Juniperus* or *Thuja*, which are currently distributed worldwide. All these species are a serious pest to economically important plants grown in commercial orchards, nursery stock, parks and gardens, and cause significant damage to cultivated and ornamental plants around the globe [34,35,36,37,38,39].

Stage-specific thermal tolerance is common in insects, especially for species with complex life stages for which specific environmental factors could lead to a variation in responses across life stages [40,41]. In contrast, hemimetabolous insects, such as aphids, where nymphs and adults live in colonies, can be subjected to the same ranges of temperature factors. It is unclear how an increase in temperature will affect the different stages of development of aphids. The aim of the study was (1) to determine the differences in the activity of antioxidant (superoxide dismutase and catalase), detoxifying (glutathione S-transferase and β-glucosidase) and oxidoreductive (peroxidase and polyphenol oxidase) enzymes of nymphs and adult aphids, (2) to determine the changes in the activity of enzymatic markers in response to the temperature increase, and (3) the correlation between metabolic activity (as a heat flow measured by microcalorimeter) and longevity of aphids was tested. We verified the hypotheses: (1) the enzymatic activity (SOD, CAT, GST, β-glucosidase, PPO and POD) of nymphs and adult aphids differ, and (2) the enzymatic defense response (changes in the activity of SOD, CAT, GST, β-glucosidase, PPO and POD) of nymphs and adult aphids differ depending on the temperature. Additionally, we expected (3) a strong positive correlation between metabolic activity and longevity, which would confirm the relationship of these characteristics of aphids.

## 2. Materials and Methods

### 2.1. Aphids

*A. pomi*, *M. rosae* and *C. cupressi* were used in the experiments and came from the stock culture of the University of Rzeszow. The aphids were grown on the same host plant species (*Chaenomeles japonica, Rosa rugosa, Juniperus scopulorum*) that were used in the experiment. The experimental aphids were bred at 16 L:8 D photoperiods, 20 ± 1 °C and 60% ± 5% humidity, and were kept in an MLR-351H climatic chamber (Sanyo Corp., Japan) under controlled conditions. Both adult wingless females and 3rd or 4th nymphs of the tested species were used for the experiments.

### 2.2. Host Plants

The host plants for *A. pomi* were seedlings of *Chaenomeles japonica*, for *M. rosae,* the host plants were seedlings of *Rosa rugosa* and for *C. cupressi* the host plants were *Juniperus scopulorum*. The plants were free of pathogens, were set in 30 cm × 30 cm × 30 cm pots (*Ch. japonica* and *R. rugosa*) or 20 cm × 20 cm × 20 cm (*J. scopulorum*), and were grown for two weeks at 20 °C to acclimatise before the experiment.

### 2.3. Effect of Temperature on the Enzymatic Activity in Nymph and Adult Aphid Tissues

The experiment was performed at three constant temperatures (20, 25 or 28 °C) under unvarying conditions, independently of each other, as previously described. Thirty adults or 30 nymphs of each species were placed on a host plant. The insects fed on the host plant for 0 (sample taken before starting the experiment), 24, 48, 72, 96 or 336 h (2 weeks). After the specified time elapsed, the aphids were harvested and frozen in liquid nitrogen. The experiments were carried out in 3 replications. Samples were stored at −85 °C (Freezer VXS 490) until analysis.

The frozen insects (30 individuals per sample) were placed in a phosphate buffer (0.1 M, pH 7.0) and homogenised at 0 °C. The resulting homogenate was centrifuged (Eppendorf Centrifuge 5810 R) at 4 °C. The supernatant obtained was used to determine enzymatic activity according to protocols described in our previous paper by Durak et al. [42] and Dampc et al. [33].

Absorbance was measured with a TECAN Infinite 200 microplate reader, and CAT was measured using a Carry 50 spectrophotometer. The wavelengths for measuring the absorbance of individual enzymes are in parentheses, incubation times are described in Dampc et al. [33].

#### 2.3.1. Antioxidant Enzymes

Superoxide dismutase (SOD) activity was measured in phosphate buffer (pH 7.8) using the method described by Wang et al. [14]. The reaction mixture contained homogenate, solution of nitro blue tetrazolium xanthine solution and xanthine oxidase solution (560 nm). Catalase activity (CAT) activity was determined using the standard method according to Aebi [43], in phosphate buffer (pH 7.0) with H_2_O_2_ solution (240 nm).

#### 2.3.2. Detoxifying Enzymes

The level of β-glucosidase was determined in phosphate buffer (pH 5.8) with p-nitrophenyl-β-d-glucopyranoside solution (400 nm) according to the reactions described by Katagiri [44]. Glutathione S-transferase (GST) activity was determined as per Leszczynski and Dixon [45] in phosphate buffer (pH 7.0) with 1-chloro-2.4-dinitrobenzene and GSH solution (340 nm).

#### 2.3.3. Oxidoreductive Enzymes

Polyphenol oxidase (PPO) activity was determined in phosphate buffer (pH 7.4) with catechol (460 nm) using the method described by Miles [46] with a slight modification from Laurema et al. [47]. Peroxidase (POD) activity was determined by the standard method according to Fehrman and Dimond [48], in phosphate buffer (pH 7.0) with pyrogallol (430).

#### 2.3.4. Protein Content in Aphid Extracts

Protein content was measured by the standard method described by Lowry et al. [49].

### 2.4. Effect of Temperature on Aphid Longevity

To determine longevity, 12 aphids of *A. pomi* and *C. cupressi* were bred separately on the host plants from birth to death, at selected temperatures (20, 25, or 28 °C). Metabolic activity of *A. pomi* and *C. cupressi* was measured at 20, 25, and 28 °C using a TAM III isothermal calorimeter equipped with TAM Assistant Software (TA Instruments). Twenty *A. pomi* aphids were placed on a young leaf and 5 *C. cupressi* were placed on a piece of juniper, then into 4.0 mL calorimetric ampoules with 0.25 μL of water. Thermal power curves were recorded over 4 h due to the rapid consumption of oxygen in the ampule by the *C. cupressi*. The metabolic activity of the aphids was calculated, as described by Dampc et al. [33]. Twelve independent repetitions for each specimen were carried out.

### 2.5. Statistical Analyses

To compare the averaged values of the enzymatic activity, we used a factorial ANOVA with statistical significance at *p* < 0.05. Averaged values of enzymatic activity were compared using three explanatory factors: temperature (20, 25 and 28 °C), time (0, 24, 48, 72, 96 and 336 h) and type of morph (nymph and adult). The analyses were performed separately for each enzyme and each aphid species. In order to reveal the links between patterns of enzyme activity, developmental stage and temperature, we analysed the enzymatic activity of nymphs and adults of three aphid species in different temperatures by principal component analysis (PCA). The PCA reduces the dimensionality of the complex enzymatic response of aphids and acquires the core of the data in a few principal components, which carry the most variation in the data. The significant role of the developmental stage and temperature in differentiating the enzymatic response along the first and second axis of PCA (hereafter, PC1 and PC2) were confirmed by a two-way ANOVA. In order to explain the pattern of aphid enzymatic response depending on developmental stage and temperature, we examined the relationships between the enzyme characteristics (individual enzyme activity and activity of groups of enzymes) and the differential in aphid’s enzymatic response represented by the PCA axis scores. For this purpose, Spearman rank correlation tests were used. To obtain the enzyme characteristics, the values for enzymes measured for each species were averaged and assigned to the following three groups: group 1— antioxidant (G1), group 2—detoxifying (G2), and group 3—oxidoreductive (G3). The Spearman correlation was also used to determine the relationships between heat flow and the longevity of aphids. All statistical analyses were done using Statistica version 13 programme (TIBCO Software Inc., 2017, http://statistica.io; accessed on 10 May 2021) and PAST 3 software.

## 3. Results

### 3.1. Antioxidant Enzyme Activity of Nymphs and Adult Aphids during Temperature Increase

The analysis of the SOD activity of the three species of aphid showed it was dependent on all examined factors: temperature, time and type of morph. The activity of SOD in *A. pomi* was significantly influenced by the temperature, time and type of morph studied (Table 1). Such relationships were also observed in *M. rosae* and *C. cupressi* (Table 1). There was also a significant interaction between temperature, time and morph type on the level of SOD activity in the studied species (Table 1, Figure S1). Significant differences in SOD activity were observed between adults and nymphs in all examined species, and the highest activity of this enzyme was found in nymphs. The analysis of CAT activity in the studied species showed its dependence on temperature, time and type of morph. All factors had a significant influence on the changes in CAT activity observed in *A. pomi*, *M. rosae* and *C. cupressi* (Table 1, Figure S1). Similarly, in the case of SOD, the significant influence of the interaction between temperature, time and type of morph was demonstrated, and was visible in all tested species *A. pomi*, *M. rosae*, and *C. cupressi* (Table 1). Significant differences were observed between the CAT activity of nymphs and adults.

### 3.2. Detoxifying Enzyme Activity of Nymphs and Adult Aphids during Temperature Increase

β-glucosidase activity was dependent on temperature, time and type of morph in *A. pomi* and *C. cupressi*. However, time did not affect the activity of this enzyme in *M. rosae*. In contrast to time, the type of morph significantly influenced the activity of this enzyme in all species. β-glucosidase activity varied between nymphs and adults in all species (Table 1, Figure S1). The GST activity of aphids was influenced by the temperature, time and type of morph in all tested species (Table 1). However, significant interactions between these three factors were only found in *M. rosae* and *C. cupressi*. We found differences in the activity of this enzyme between different morphs (Table 1, Figure S1).

### 3.3. Oxidoreductive Enzyme Activity of Nymphs and Adult Aphids during Temperature Increase

POD activity was significantly dependent on the three factors: temperature, time and morph type in all the studied species, but a significant influence of the interaction between temperature, time and type of morph was observed in *M. rosae* and *C. cupressi* (Table 1, Figure S1). In all tested aphid species, PPO activity was significantly dependent on temperature, time and morph type (Table 1, Figure S1). We also observed significant interactions between these factors in individual species.

### 3.4. Enzymatic Differences between Nymphs and Adult Aphids during Temperature Increase

**Figure 1:**
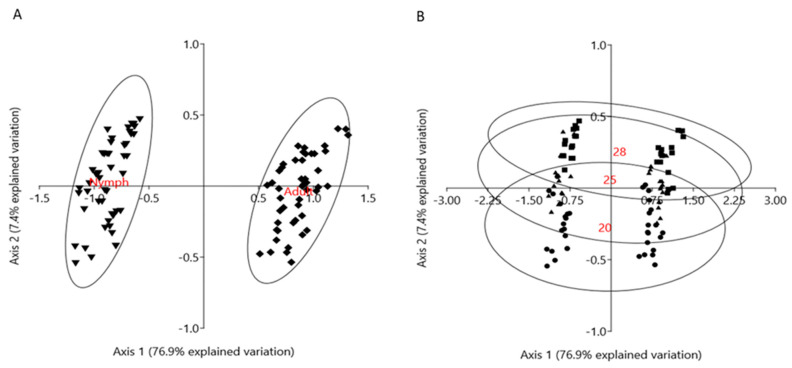
Principal component analysis (PCA) of enzyme data from three aphid species. (**A**) The first axis (PC1) segregated the enzyme data according to aphid developmental stage, and to a lesser extent, according to temperature (inverted triangle—nymphs, diamond—adults). (**B**) The second axis (PC2) segregated the enzyme data according to temperature (dot—20 °C, triangle—25 °C, square—28 °C). The groups are marked with 95% ellipses.

**Figure 2:**
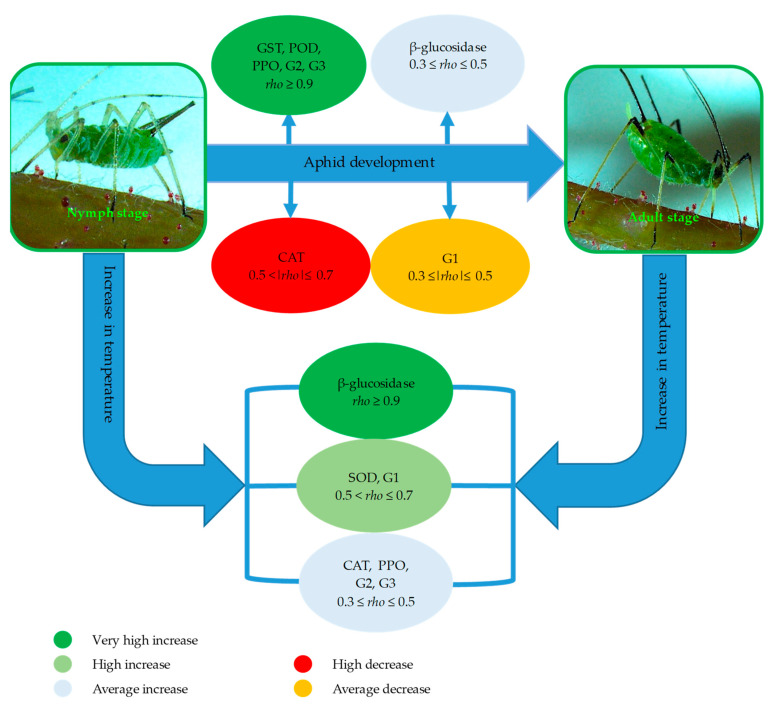
Schematic diagram of the main changes in aphid’s enzyme activity in response to course of development and temperature. G1, G2 and G3 groups of enzymes: antioxidant, detoxifying and oxidoreductive, respectively. Enzyme activity responses were ranked based on Spearman’s *rho* correlation coefficients given in Table 3. Correlation coefficients with values indicating at least an average correlation (0.3 ≤ *rho*) were taken into consideration. See Table 3 for detailed results.

The PCA of all investigated enzymes in the tissues of three species of aphids (*Aphis pomi*, *Macrosiphum rosae* and *Cinara cupressi*) is presented in Figure 1. The first two PCA axes covered 84.33% of the variance in the data. The first PCA axis (which explained 76.9% of the variation), with a high probability, separated samples according to the developmental stage (type of morph) and to a lesser extent, temperature. This was supported by a two-way ANOVA test that confirmed the dominant role of the developmental stage, and the subdominant role of temperature in explaining enzymatic variability (Table 2). Moreover, PC1 had a very strong, positive correlation with enzymes: GST, POD, PPO and groups of enzymes: G2 and G3 (detoxifying and oxidoreductive), as well as a strong, negative correlation with CAT (Table 3, Figure 2). The second PCA axis explained only 7.4% of the variation and segregated the samples mainly according to the temperature change and, in a smaller proportion, according to the developmental stage (Table 2). PC2 had a strong, positive correlation with enzymes: SOD and β-glucosidase, as well as a group of enzymes, G1 (antioxidant) (Table 3, Figure 2). This analysis showed a clear separation of the enzyme activities of nymphs and adult aphids, and also the physiological defense of nymphs and adult aphids against temperature stress. The main role in the enzymatic defense response of nymphs is played by antioxidant enzymes (G1) (Figure 1A), the activity of which is correlated with the type of morph (nymph), and also had a strong correlation with temperature (Table 3, Figure 2). The detoxifying (G2) and oxidoreductive (G3) enzymes showed a higher correlation with type of morphs—adults (Figure 1A, Table 3, Figure 2). The defense responses of both nymphs and adults were dependent on temperature (Figure 1B). The enzymatic activity increased with increasing temperature and was clearly higher at 28 °C than at 20 °C for both nymph and adults (Figure 1B).

### 3.5. Effect of Temperature on the Longevity of Aphids

Changes were found in the metabolic heat of *M. rosae* and *C. cupressi* related to temperature increase, and these changes negatively impacted the lifespan of the aphids. Spearman’s rank correlation coefficient values were *rho* = −0.62, (*p* < 0.001) for *M. rosae* and *rho* = −0.71 for *C. cupressi* (*p* < 0.001), and confirmed the strong negative correlation between heat flow and longevity.

## 4. Discussion

The effects of stress caused by temperature increase on insects may depend on many factors, such as frequency, amplitude, duration of the stress, sex, or developmental stage of the insect [8,50,51]. In aphids, due to their relatively short development period and colony life, all development stages may be exposed to stress caused by temperatures exceeding their thermal optimum. The activity of the tested enzymes in the three aphid species increased, which suggests the involvement of these enzymes in reducing the adverse effects of ROS generated by thermal stress and the defensive function (Figure 1, Figure S1).

Our research has shown that the enzymatic activity of nymphs and adults differ from each other (Figure 1A). The enzymatic defense response varied depending on the temperature (Figure 1B). The results of our study demonstrate that the antioxidant enzyme system is more efficient in aphid nymphs and provides them with quick and effective protection against stress factors. At the same time, oxidoreductive and detoxifying enzymes are more active in adults. This could suggest that the nymphs’ system of detoxifying and oxidoreductive enzymes is less efficient, and hence, nymphs may be less effective in degrading toxic substances. The efficient operation of enzyme groups provides nymphs with effective protection against stress. The high activity of antioxidant enzymes enables the nymphs to mature under stressful conditions, such as high temperatures (Figure 1).

Stage-specific thermal tolerance is very common in insects [41], but the larvae of different orders of insects respond to stress differently. Some authors have shown that the enzymatic activity of larvae is higher than that of adults. The potential role of antioxidant enzymes in preventing damage from abiotic stresses during growth in the third to sixth-instar larval stages was noted in larvae of Lepidoptera, and high stage-specific antioxidative enzyme activity was also reported in, for example, *Chilo suppressalis*. For the larvae of Lepidoptera, upregulation of CsCAT enhanced the defense response of *C. suppressalis* by weakening the effects of environmental stresses. CAT was expressed highly in the third to sixth-instar larval stages, which supports the potential role of the enzyme in preventing damage from abiotic stresses during the developmental process [52]. In addition, activity of this enzyme was higher in larvae, as a response to temperature stress. Stage-specific antioxidative enzyme activity related to the sensitivity of stages to heat stress was also characteristic for beetles *Ophraella communa* [8]. Furthermore, in honeybees *Apis mellifera*, SOD activity was higher in larvae than in the adult stage [53]. In contrast, high enzyme activity levels in the adult of *Osmia bicornis* were related, which was explained by intensified mitochondrial activity due to high energy demands at this stage, especially during flight because the flight muscles are exposed to high levels of ROS [23,54]. Also, Krishnan et al. [15,24] noted a higher antioxidant potential in adults of *Leptinotarsa decemlineata* than in the larval stage. Thus, these insects were able to adapt quickly to increased oxidative stress arising from plant prooxidant allelochemicals by regulation of antioxidant enzyme activities [15]. Previous studies have shown that the antioxidant mechanism of aphids depends on the host plant; differences were not only observed between aphid species, but also between populations of the same species that inhabited different hosts [19].

The increase in temperature positively influenced the aphid metabolism, causing an increase in heat energy. As a consequence, aphids living in higher temperatures had shorter longevity. Temperature is one factor widely discussed as affecting longevity and it can determine the lifespan of many model organisms, for example, fungi *Saccharomyces cerevisiae* [55], *Caenorhabditis elegans* [56] or *Drosophila melanogaster* [32]. For aphids, it has also been noted that there is a negative correlation between temperature and longevity [57,58,59], but in some aphids, the opposite effect was observed in relation to the optimum temperature of the species [60]. Our research confirmed that metabolic rate is an important determinant of longevity, thus indicating that an increase in ambient temperature will increase enzyme activity and shorten aphid longevity.

Our research shows that the effectiveness of aphid antioxidant, detoxifying and oxidoreductive mechanisms depends on many factors, such as the species of aphid, temperature, time and also type of morph. It seems that sap-feeding nymphs of aphids, show great adaptability due to their enzyme system, which is especially important in adaptation to host plants (in the case of generalists) as well as abiotic factors (temperature). Our results strongly correspond to the data obtained for the English grain aphid *Sitobion avenae*, which showed that the basal thermal tolerance of this species varied during development and was highest in the 3rd and 4th nymphs [61]. Changes in basal tolerance at the adult stage might result from a trade-off between heat tolerance and reproductive output, but may also result from behavioural thermoregulation abilities present in adult aphids [59]. Probably because the behavioural mechanisms of nymphs function poorly, we observed the need for high antioxidant activity of enzymes to effectively enable the protection of nymphs and allow them to continue development to maturity.

## 5. Conclusions

The enzymatic activity of aphids is mainly determined by the type of morph. The increase in temperature causes changes in enzyme activity, which is highest at 28 °C in both nymphs and adults. Nymphs are characterised by an increased share of antioxidant enzymes, while in adults, detoxification and oxidoreductive enzymes dominate. An increase in energy activity caused by an increase in temperature will reduce the longevity of aphids.

## Figures and Tables

**Table 1:** Analysis of enzymatic activity in the tissues of three species of aphid: *Aphis pomi*, *Macrosiphum rosae* and *Cinara cupressi*. ANOVA was used to test differences between average enzymatic activities in different conditions (*p* < 0.05). (T—temperature, t—time, m—type of morph).

	SOD	CAT	GST	β-Glucosidase	POD	PPO
*Aphis pomi*						
T	F_(2,72)_ = 4.99 ***	F_(2,72)_ = 72.42 ***	F_(2,72)_ = 20.61 ***	F_(2,72)_ = 534.30 ***	F_(2,72)_ = 31.95 ***	F_(2,72)_ = 64.17 ***
t	F_(5,72)_ = 9.96 ***	F_(5,72)_ = 114.14 ***	F_(5,72)_ = 6.42 ***	F_(5,72)_ = 474.83 ***	F_(5,72)_ = 26.70 ***	F_(5,72)_ = 64.61 ***
m	F_(1,72)_ = 2937.76 ***	F_(1,72)_ = 677.21 ***	F_(1,72)_ = 555,58 ***	F_(1,72)_ = 14.82 ***	F_(1,72)_ = 970.45 ***	F_(1,72)_ = 13.02 ***
T × m	F_(2,72)_ = 3.63 ***	F_(2,72)_ = 32.87 ***	F_(2,72)_ = 4.31 *	F_(2,72)_ = 0.18	F_(2,72)_ = 2.01	F_(2,72)_ = 11.56 ***
T × t	F_(10,72)_ = 1.72	F_(10,72)_ = 20.21 ***	F_(10,72)_ = 1.85	F_(10,72)_ = 54.52 ***	F_(10,72)_ = 3.58 ***	F_(10,72)_ = 5.62 ***
t × m	F_(5,72)_ = 2.87 **	F_(5,72)_ = 20.51 ***	F_(5,72)_ = 2.83 *	F_(5,72)_ = 4.33 ***	F_(5,72)_ = 5.28 ***	F_(5,72)_ = 14.41 ***
T × t × m	F_(10,72)_ = 1.90 *	F_(10,72)_ = 6.29 ***	F_(10,72)_ = 1.07	F_(10,72)_ = 4.30 ***	F_(10,72)_ = 1.59	F_(10,72)_ = 26.65 ***
*Macrosiphum rosae*						
T	F_(2,72)_ = 63.86 ***	F_(2,72)_ = 2.33 **	F_(2,72)_ = 5.39 **	F_(2,72)_ = 7.66 ***	F_(2,72)_ = 6.25 **	F_(2,72)_ = 15.98 ***
t	F_(5,72)_ = 43.43 ***	F_(5,72)_ = 32.83 ***	F_(5,72)_ = 2.86 *	F_(5,72)_ = 1.39	F_(5,72)_ = 19.10 ***	F_(5,72)_ = 3.98 **
m	F_(1,72)_ = 976.55 ***	F_(1,72)_ = 37.78 ***	F_(1,72)_ = 7884.39 ***	F_(1,72)_ = 20.58 ***	F_(1,72)_ = 2351.88 ***	F_(1,72)_ = 108.12***
T × m	F_(2,72)_ = 84.61 ***	F_(2,72)_ = 0.43 **	F_(2,72)_ = 0.10	F_(2,72)_ = 1.17	F_(2,72)_ = 4.24 *	F_(2,72)_ = 0.14
T × t	F_(10,72)_ = 15.83 ***	F_(10,72)_ = 2.18 **	F_(10,72)_ = 2.71 **	F_(10,72)_ = 1.62	F_(10,72)_ = 2.21 *	F_(10,72)_ = 2.43 *
t × m	F_(5,72)_ = 34.01 ***	F_(5,72)_ = 10.28 ***	F(_5,72)_ = 2.09	F_(5,72)_ = 3.22 *	F_(5,72)_ = 17.58 ***	F_(5,72)_ = 3.55 **
T × t × m	F_(10,72)_ = 14.17 ***	F_(10,72)_ = 3.81 ***	F_(10,72)_ = 2.68 **	F_(10,72)_ = 1.27	F_(10,72)_ = 2.33 *	F_(10,72)_ = 3.03 **
*Cinara cupressi*						
T	F_(2,72)_ = 118.67 ***	F_(2,72)_ = 335.74 ***	F_(2,72)_ = 154.28 ***	F_(2,72)_ = 13.74 ***	F_(2,72)_ = 114.34 ***	F_(2,72)_ = 60.89 ***
t	F_(5,72)_ = 17.43 ***	F_(5,72)_ = 529.52 ***	F_(5,72)_ = 85.47 ***	F_(5,72)_ = 6.00 ***	F_(5,72)_ = 60.27 ***	F_(5,72)_ = 12.05 ***
m	F_(1,72)_ = 1729.37 ***	F_(1,72)_ = 378.08 ***	F_(1,72)_ = 8183.41 ***	F_(1,72)_ = 2467.09 ***	F_(1,72)_ = 2661.945 ***	F_(1,72)_ = 2297.45 ***
T × m	F_(2,72)_ = 24.44 ***	F_(2,72)_ = 123.77 ***	F_(2,72)_ = 21.18 ***	F_(2,72)_ = 6.37 **	F_(2,72)_ = 124.26 ***	F_(2,72)_ = 37.06 ***
T × t	F_(10,72)_ = 7.57 ***	F_(10,72)_ = 114.84 ***	F_(10,72)_ = 9.08 ***	F_(10,72)_ = 4.07 ***	F_(10,72)_ = 15.27 ***	F_(10,72)_ = 19.35 ***
t × m	F_(5,72)_ = 7.67 ***	F_(5,72)_ = 173.21 ***	F_(5,72)_ = 48.55 ***	F_(5,72)_ = 3.16 *	F_(5,72)_ = 53.85 ***	F_(5,72)_ = 17.96 ***
T × t × m	F_(10,72)_ = 3.17 **	F_(10,72)_ = 53.65 ***	F_(10,72)_ = 3.46 ***	F_(10,72)_ = 1.72	F_(10,72)_ = 13.71 ***	F_(10,72)_ = 11.26 ***

* *p* < 0.05; ** *p* < 0.01; *** *p* < 0.001.

**Table 2:** ANOVA test results confirm that the first component (PC1) mainly shows that the strong differentiation of aphid enzymatic response depends on developmental stage (type of morph: nymphs, adults), and the second component (PC2) mainly shows that the differentiation of aphid enzymatic response depends on temperature.

	Test Score (F)
PC1	
Temperature (T)	38.31 ***
Morph (m)	4635.07 ***
T × m	3.31 *
PC2	
Temperature (T)	96.06 ***
Morph (m)	4.32 *
T × m	0.85

* *p* < 0.05; *** *p* < 0.001.

**Table 3:** Correlation between enzyme data from aphid species (individual enzymes and specific groups of enzymes) and PCA axes that represent changes in enzyme data depending on aphid developmental stage and temperature (Spearman rank correlation test).

	Spearman’s *rho* Correlation Coefficients	
	PC1	PC2
Enzymes		
SOD	−0.27 **	0.64 ***
CAT	−0.55 ***	0.37 ***
β-glucosidase	0.37 ***	0.88 ***
GST	0.88 ***	0.24 *
POD	0.90 ***	0.12
PPO	0.95 ***	0.30 **
Groups of enzymes		
G1 (antioxidant enzymes)	−0.37 ***	0.65 ***
G2 (detoxifying enzymes)	0.90 ***	0.35 ***
G3 (oxidoreductive enzymes)	0.97 ***	0.30 **

* *p* < 0.05; ** *p* < 0.01; *** *p* < 0.001.

## Data Availability

The data presented in this study are available in the article. Additional data are available on request from the corresponding author.

## Supplementary Materials

The following are available online at https://www.mdpi.com/article/10.3390/antiox10081181/s1, Figure S1: Principle component analysis (PCA) of enzymes data set of three aphid species. The groups are marked with 95% ellipses.
