# Supplementary material for: Changes in Antioxidative, Oxidoreductive and Detoxification Enzymes during Development of Aphids and Temperature Increase
Source: Antioxidants (Basel). 2021 Jul 25;10(8):1181. doi: 10.3390/antiox10081181 (PMC8388978; doi:10.3390/antiox10081181)
Supplement: Supplementary file 1 [file antioxidants-10-01181-s001.zip › antioxidants-1282941-supplementary.pdf]

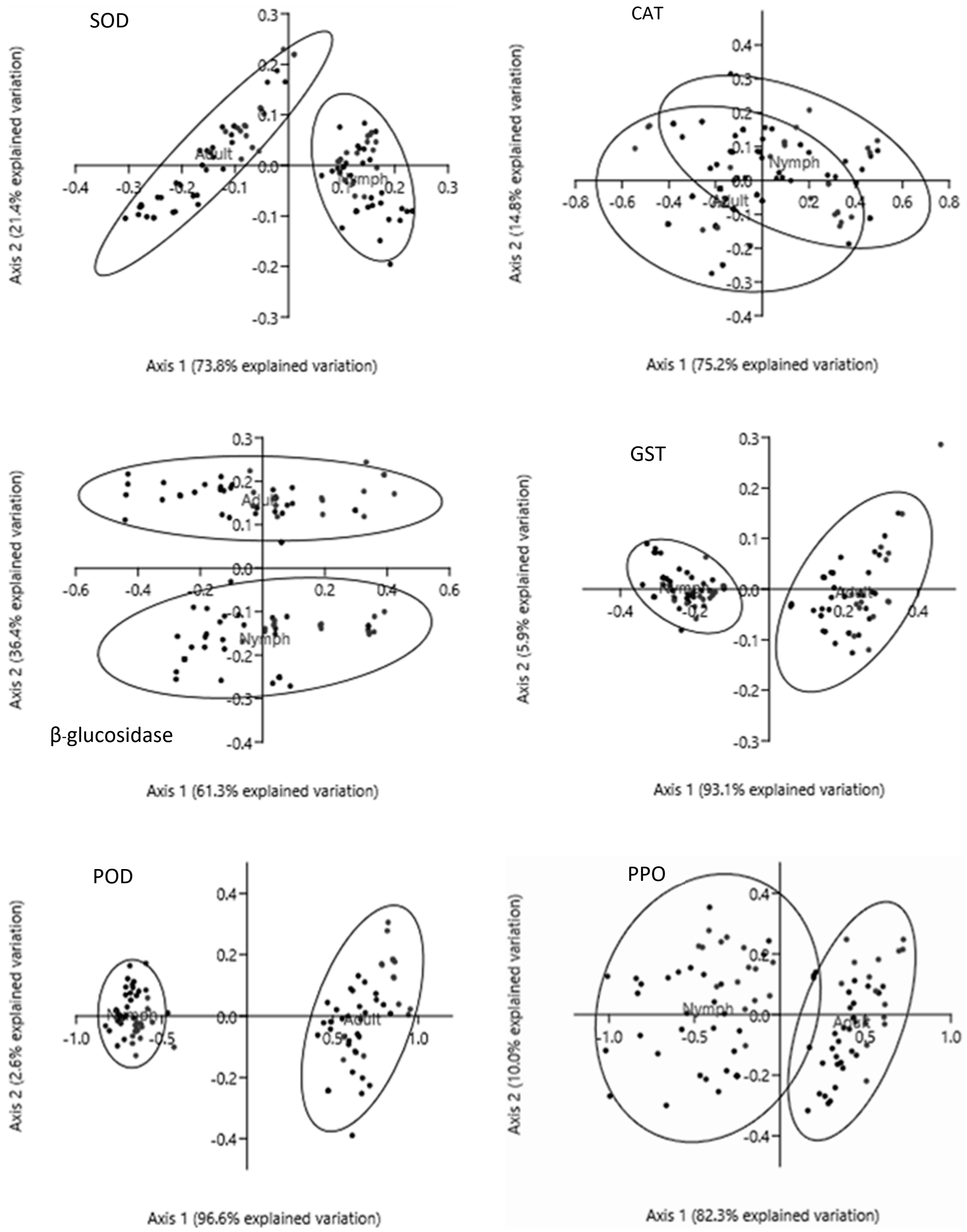

Figure S1. Principle component analysis (PCA) of enzymes data set of three aphid species. The groups are marked with 95% ellipses.
